# Supplementary material for: Associations between Brain Microstructure and Phonological Processing Ability in Preschool Children
Source: Children (Basel). 2022 May 26;9(6):782. doi: 10.3390/children9060782 (PMC9221994; doi:10.3390/children9060782)
Supplement: Supplementary file 1 [file children-09-00782-s001.zip › children-1693484-supplementary.pdf]

Supplement:

Zhou et al., Associations between Brain Microstructure and Language Ability in Preschool Children

Supplementary Table S1 Mediation analysis results on DTI Measures

(A) FA_Age+PPRS+

With ID, Sex, Handness, Mom education as covariates

	Path a	Path b	Path c	Path c’	Mediation
	(X→M)	(M→Y)	(X→Y)	(X→Y)	Path (c-c’)
Model 1: X (Age)→Y (PPRS) mediated by M (FA_Age+PPRS+)					
β	0.023	77.872	4.234	2.451	1.784
p	0.000	0.000	0.000	0.011	0.000
Model 2: X (Age)→Y (FA_Age+PPRS+) mediated by M (PPRS)					
β	4.234	0.002	0.023	0.016	0.007
p	0.000	0.057	0.000	0.000	0.010
Model 3: X (PPRS)→Y (Age) mediated by M (FA_Age+PPRS+)					
β	0.004	18.114	0.123	0.058	0.065
p	0.053	0.000	0.000	0.000	0.000
Model 4: X (PPRS)→Y(FA_Age+PPRS+) mediated by M (Age)					
β	0.123	0.016	0.004	0.002	0.002
p	0.000	0.000	0.053	0.057	0.000
Model 5: X (FA_Age+PPRS+)→Y(PPRS) mediated by M (Age)					
β	26.351	2.451	142.452	77.872	64.580
p	0.000	0.011	0.000	0.000	0.000
Model 6: X (FA_Age+PPRS+)→Y(Age) mediated by M (PPRS)					
β	142.452	0.058	26.351	18.114	8.237
p	0.000	0.000	0.000	0.000	0.000

(B) MD_Age-PPRS-

With ID, Sex, Handness, Mom education as covariates

	Path a	Path b	Path c	Path c’	Mediation
	(X→M)	(M→Y)	(X→Y)	(X→Y)	Path (c-c’)
Model 1: X (Age)→Y (PPRS) mediated by M (MD_Age-PPRS-)					
β	-0.021	-31.663	4.234	3.579	0.655
p	0.000	0.030	0.000	0.000	0.037
Model 2: X (Age)→Y (MD_Age-PPRS-) mediated by M (PPRS)					

β	4.234	-0.001	-0.021	-0.018	-0.003
p	0.000	0.064	0.000	0.000	0.078
Model 3: X (PPRS)→Y (Age) mediated by M (MD_Age-PPRS-)					
β	-0.003	-16.376	0.123	0.076	0.047
p	0.062	0.000	0.000	0.000	0.000
Model 4: X (PPRS)→Y(MD_Age-PPRS-) mediated by M (Age)					
β	0.123	-0.018	-0.003	-0.001	-0.002
p	0.000	0.000	0.062	0.064	0.000
Model 5: X (MD_Age-PPRS-)→Y(PPRS) mediated by M (Age)					
β	-25.737	3.579	-123.781	31.663	-92.118
p	0.000	0.000	0.000	0.030	0.000
Model 6: X (MD_Age-PPRS-)→Y(Age) mediated by M (PPRS)					
β	-123.781	0.076	-25.737	-16.376	-9.362
p	0.000	0.000	0.000	0.000	0.000

Supplementary Table S2 Mediation analysis results on FN Characteristics.

(A) Net1 Number of fiber between CUN.R and CAL.R

With ID, Sex, Handness, Mom education as covariates

	Path a	Path b	Path c	Path c'	Mediation
	(X→M)	(M→Y)	(X→Y)	(X→Y)	Path (c-c')
Model 1: X (Age)→Y (PPRS) mediated by M (Net1)					
β	51.620	0.004	4.234	4.016	0.218
p	0.000	0.077	0.000	0.000	0.106

(B) Net2 Number of fiber between CAL.R and LING.R

With ID, Sex, Handness, Mom education as covariates

	Path a	Path b	Path c	Path c'	Mediation
	(X→M)	(M→Y)	(X→Y)	(X→Y)	Path (c-c')
Model 1: X (Age)→Y (PPRS) mediated by M (Net2)					
β	39.063	0.004	4.234	4.090	0.145
p	0.000	0.124	0.000	0.000	0.181

(C) Net3 Number of fiber between SOG.R and IOG.R

With ID, Sex, Handness, Mom education as covariates

	Path a	Path b	Path c	Path c'	Mediation
	(X→M)	(M→Y)	(X→Y)	(X→Y)	Path (c-c')
Model 1: X (Age)→Y (PPRS) mediated by M (Net3)					
β	21.229	0.007	4.234	4.088	0.147

p	0.002	0.098	0.000	0.000	0.145

(D) Net4 Number of fiber between IPL.R and SMG.R

With ID, Sex, Handness, Mom education as covariates

	Path a	Path b	Path c	Path c’	Mediation
	(X→M)	(M→Y)	(X→Y)	(X→Y)	Path (c-c’)
Model 1: X (Age)→Y (PPRS) mediated by M (Net4)					
β	61.424	0.004	4.234	3.976	0.258
p	0.000	0.004	0.000	0.011	0.016
Model 2: X (Age)→Y (Net4) mediated by M (PPRS)					
β	4.234	7.556	61.424	29.428	31.996
p	0.000	0.051	0.000	0.073	0.004
Model 3: X (PPRS)→Y (Age) mediated by M (Net4)					
β	11.174	0.000	0.123	0.118	0.005
p	0.000	0.086	0.000	0.000	0.118
Model 4: X (PPRS)→Y(Net4) mediated by M (Age)					
β	0.123	29.428	11.174	7.556	3.618
p	0.000	0.073	0.000	0.051	0.077
Model 5: X (Net4)→Y(PPRS) mediated by M (Age)					
β	0.002	3.976	0.011	0.004	0.007
p	0.063	0.011	0.000	0.004	0.000
Model 6: X (Net4)→Y(Age) mediated by M (PPRS)					
β	0.011	0.118	0.002	0.000	0.001
p	0.000	0.000	0.063	0.086	0.000

(E) Net5 Number of fiber between PAL.R and OLF.L

With ID, Sex, Handness, Mom education as covariates

	Path a	Path b	Path c	Path c’	Mediation
	(X→M)	(M→Y)	(X→Y)	(X→Y)	Path (c-c’)
Model 1: X (Age)→Y (PPRS) mediated by M (Net5)					
β	3.488	0.030	4.234	4.130	0.104
p	0.000	0.387	0.000	0.000	0.414

(F) Net6 Number of fiber between INS.R and TPOmid.R

With ID, Sex, Handness, Mom education as covariates

	Path a	Path b	Path c	Path c’	Mediation
	(X→M)	(M→Y)	(X→Y)	(X→Y)	Path (c-c’)
Model 1: X (Age)→Y (PPRS) mediated by M (Net6)					

β	42.528	0.003	4.234	4.106	0.128
p	0.000	0.151	0.000	0.000	0.177
